# Altered cerebral benzodiazepine receptor binding in post-traumatic stress disorder

**DOI:** 10.1038/s41398-018-0257-9

**Published:** 2018-10-04

**Authors:** Inbal Reuveni, Allison C. Nugent, Jessica Gill, Meena Vythilingam, Paul J. Carlson, Alicja Lerner, Alexander Neumeister, Dennis S. Charney, Wayne C. Drevets, Omer Bonne

**Affiliations:** 1 Department of Psychiatry, Hadassah Hebrew University Medical Center, Jerusalem, Israel; 2 Experimental Therapeutics and Pathophysiology Branch, National Institute of Mental Health, National Institutes of Health, Bethesda, MD USA; 3 Center for Neuroscience and Regenerative Medicine (CNRM), National Institutes of Health, Bethesda, MD USA; 4 Deployment Health Clinical Center, Defense Centers of Excellence for Psychological Health and Traumatic Brain Injury, Silver Spring, MD USA; 5 Department of Psychiatry, University of Utah School of Medicine, Salt Lake City, UT USA; 6 Controlled Substance Staff, Center for Drug Evaluation and Research, Food and Drug Administration, Silver Spring, MD USA; 7 Institute of Mental Health Research (IMHR), University of Ottawa, Ottawa, ON Canada; 8 Mood and Anxiety Disorders Program, Department of Psychiatry, Icahn School of Medicine at Mount Sinai, New York, NY USA; 9 Janssen Research and Development, LLC of Johnson & Johnson Inc., Titusville, NJ USA

## Abstract

Agonists of the γ-aminobutyric acid (GABA) type A benzodiazepine (BZD) receptor exert anxiolytic effects in anxiety disorders, raising the possibility that altered GABA-ergic function may play a role in the pathophysiology of anxiety disorders, such as post-traumatic stress disorder (PTSD). However, few neuroimaging studies have assessed the function or binding potential of the central GABA_A_ BZD receptor system in PTSD. Therefore, our aim was to compare the BZD receptor binding potential between PTSD patients and healthy controls. Twelve medication-free participants with a current diagnosis of PTSD and 15 matched healthy controls underwent positron emission tomography (PET) imaging using [^11^C] flumazenil. Structural magnetic resonance imaging (MRI) scans were obtained and co-registered to the PET images to permit co-location of neuroanatomical structures in the lower resolution PET image data. Compared to healthy controls, PTSD patients exhibited increased BZD binding in the caudal anterior cingulate cortex and precuneus (*p*’s < 0.05). Severity of PTSD symptoms positively correlated with BZD binding in the left mid- and anterior insular cortices. This study extends previous findings by suggesting that central BZD receptor system involvement in PTSD includes portions of the default mode and salience networks, along with insular regions that support interoception and autonomic arousal.

## Introduction

γ-Aminobutyric acid (GABA) is the principle inhibitory neurotransmitter in the brain. Agonists of the GABA-subtype A benzodiazepine (BZD) receptor complex constitute one of the major classes of compounds used to treat anxiety disorders, including post-traumatic stress disorder (PTSD). Nevertheless, only a few studies have examined cerebral BZD receptor binding and/or GABA concentrations in PTSD, were limited to combat exposed males, and proved ambiguous in their findings. Thus, reduced BZD receptor binding potential (BP) in the prefrontal cortex was initially reported in combat-trauma veterans with PTSD compared to healthy controls in a single-photon emission computed tomography (SPECT)-[^123^I]iomazenil study^1^ while widespread reductions in cortical regions, hippocampus, and thalamus were reported by a positron emission tomography (PET) evaluation^2^ in a similar population. In contrast, no difference was observed between subjects with PTSD and controls in a SPECT-[^123^I]Iomazenil study of Gulf War veterans^3^. The sample sizes in these studies were small, and the differential effects of current or past psychotropic medications across these studies may also have contributed to their variable results (see Discussion).

Other measures examined have also implicated altered central GABA-ergic function in PTSD. In civilians with PTSD, greater cortical excitability was observed after transcranial magnetic stimulation, suggesting widespread impairment of GABAergic function^4^. Magnetic resonance spectroscopy (MRS) studies of cerebral GABA concentrations showed lower levels of GABA in PTSD patients in parieto-occipital and temporal cortices in one study^5^, whereas higher GABA levels in PTSD patients in the dorsolateral prefrontal cortex and anterior cingulate cortex (ACC) were found in another^6^. In a third study, GABA levels were significantly lower in PTSD subjects than in controls in the insula, while GABA did not differ between groups in the dorsal ACC^7^. A meta-analysis of ^1^H-MRS studies measuring GABA levels across psychiatric disorders did not show significant differences in GABA levels in PTSD patients compared to controls^8^. Nevertheless, the interpretation of these findings is still of some controversy, with some authors concluding that primarily extracellular GABA contributes to the MRS signal^9^, while others conclude that largely intracellular GABA is measured^10^.

### Aims of study

Using PET and [^11^C]flumazenil we explored differences in the BZD receptor BP between male and female PTSD patients with civilian trauma, naïve to psychotropic medication, without current or past substance abuse and healthy non-traumatized controls.

## Methods

### Subjects

Twelve medication-free outpatients with PTSD (33.4 ± 10.2 years old, 7 women) and 15 non-traumatized, healthy subjects (34.8 ± 10.4 years old, 8 women) were recruited to the study through advertisements in NIMH and local media (we had originally intended to recruit 15 participants per group, see Data analysis section). Participants provided written informed consent prior to participation and received monetary compensation for participation. Psychiatric diagnoses were determined using the Structured Clinical Interview for DSM-IV (SCID)^11^. Severity of PTSD was determined using the Clinician-Administered PTSD Scale (CAPS)^12^ and that of depressive and anxiety symptoms was assessed using the Inventory of Depressive Symptomatology^13^ and Hamilton Anxiety Rating Scale^14^. Patients suffered pre-pubertal (*n* = 5) or adult (*n* = 7) civilian trauma. Time elapsed from exposure to trauma was 22 ± 4 years in patients exposed to pre-pubertal trauma, and 9.1 ± 8.8 years in patients with adult exposure. Participants underwent physical and laboratory examinations and did not meet criteria for current or past alcohol or substance abuse or dependence. PTSD patients were naïve to psychotropic medication. Control subjects had no personal or family history (in first-degree relatives) of psychiatric disorders.

This study was performed within the National Institute of Mental Health **(**NIMH) intramural program between March 2004 and June 2005 and approved by the NIMH institutional review board.

### Positron emission tomography

PET scans were acquired with subjects at rest with eyes closed using a GE Advance scanner with septa retracted (35 contiguous slices; 4.25 mm plane separation; reconstructed 3D spatial resolution = 6–7 mm full-width at half-maximum). A transmission scan was acquired to correct for attenuation. Following transmission scanning, a dose of approximately 20 mCi of high specific activity [^11^C]-flumazenil was injected. The upper limit to the injection mass of [^11^C]-flumazenil was set at 9 µg per 70 kg in all studies. A 60-min dynamic emission image of the brain was initiated at injection. Subject motion correction during the PET acquisition was performed with a mutual information registration of each scan time-frame to a standard frame before attenuation correction. Based on the calculated motion, the transmission images were re-sliced and projected for final reconstruction and realignment. To provide an anatomical framework for analysis of the PET images, structural magnetic resonance imaging (MRI) scans were acquired using a 3.0 Tesla scanner (Signa; GE Medical Systems, Waukesha, WI) and a T1-weighted pulse sequence (MP-RAGE; voxel size = 0.9 × 0.9 × 1.2 mm). PET images were registered to the individual’s MRI with a mutual information algorithm.

### Data analysis

#### Power analysis

At the time the study was conceived, the most similar available study was the SPECT-[^123I^]iomazenil study reported Bremner et al.^1^, comparing patients with Vietnam combat-related PTSD and healthy control subjects. Normalized results from this study were a mean of 31.0 (SD = 10.1) in the PTSD patients versus a mean of 52.7 (SD = 15.0) for healthy comparison subjects. Based upon the magnitude of this difference between groups, and the within-group standard deviations, sample sizes of 15 participants per group provide very high power (>0.99) to detect a difference in regional BZD receptor binding between groups for *α* = 0.05 (two-tailed). Since we encountered difficulties in recruitment of patients with PTSD who agreed to perform the PET procedure, we recalculated our power analysis for groups of 15 healthy control subjects and 12 participants with PTSD. This revealed that incorporating groups of this size still provided power in excess of 0.95 to detect a difference in regional BZD receptor binding between groups for *α* = 0.05 (two-tailed). Therefore, recruitment was study with the PTSD patient group containing 12 participants.

#### Image analysis

BP images were created using the two-step version of the simplified reference tissue model (SRTM2)^15^. Input kinetics for the reference tissue were derived from the pons (delimited on each subject’s MR image), where [^11^C]flumazenil binding predominantly reflects free and nonspecifically bound radiotracer^16^. Simplified reference tissue modeling approaches with the pons as the reference tissue were validated against more invasive approaches that used arterial plasma input functions for deriving [^11^C]flumazenil BP. The BP values obtained using these two approaches were highly correlated (*r* = 0.96 to *r* = 1.00)^16,17^. The MRI images (to which the PET data were co-registered) also were used to transform the BP images to a common spatial array (the Montreal Neurological Institute [MNI] template) using SPM2 software (Wellcome Department of Imaging Neuroscience, UCL, London, UK). The PET images then were filtered using a 10-mm Gaussian smoothing kernel to compensate for the effects of potential misalignment error arising during spatial normalization and individual anatomical variation.

[^11^C]-Flumazenil BP values were compared between groups in a voxel-wise analysis using a two-sample *t*-test model. Regional between-group differences in the mean BP were considered significant if the peak voxel *t*-value corresponded to *p*_uncorrected_ ≤ 0.005 and the cluster-level *p*-value (for clusters of contiguous, similarly valenced *t*-values corresponding to *p*_uncorrected_ < 0.005) remained significant after applying corrections for multiple testing using the “cluster test”^18^. The coordinates of each voxel were converted to the stereotaxic array of Talairach and Tournoux^19^ using a linear transformation (http://imaging.mrc-cbu.cam.ac.uk/downloads/MNI2tal/mni2tal.m).

To assess the relationship between illness severity and BZD receptor binding, we performed a correlational analysis post hoc which assessed the association between the CAPS score and the regional [^11^C]flumazenil BP. Due to the exploratory nature of this analysis we applied the same significance threshold as for the group-level comparisons (i.e., peak voxel *t*-value corresponding to *p*_uncorrected_ ≤ 0.005) without applying corrections for multiple testing.

## Results

Patients and controls were similar in sociodemographic measures but differed significantly in PTSD, anxiety, and depression severity ratings (see Table 1). No significant difference was found in any sociodemographic or behavioral measures between patients exposed to pre-pubertal versus adult trauma (data available on request).

**Table 1:** Sociodemographic and clinical variables of subjects with PTSD and healthy controls

	PTSD (*n* = 12)	Healthy Control (*n* = 15)		
	Mean (s.d.)	Mean (s.d.)	*t*	*p*-Value
Age	34.8 (10.2)	33.8 (10.5)	0.26	.80
CAPS	64.3 (13.8)	0.00 (0.00)	17.5	.00
HAM-A	13.4 (8.33)	0.54 (0.97)	5.55	.00
IDS	20.8 (11.96)	0.85 (0.99)	5.99	.00
I.Q.	111.3 (8.95)	114.5 (8.26)	0.96	.35
	*N*	*N*	*χ* ^2^	*p*-Value
Smoking (Yes/No)	1/11	1/14	0.9	0.81
Gender (Female/Male)	7/5	8/7	1.2	0.67
Race (Caucasian/African-American/Hispanic)	9/2/1	11/3/1	1.0	0.72

*PTSD* post-traumatic stress disorder, *CAPS* Clinician-Administered PTSD Scale, *HAM-A,* Hamilton Anxiety Rating Scale, *IDS* Inventory of Depressive Symptomatology, *MDD*, major depressive disorder, *SD*, standard deviation

**Fig. 1:**
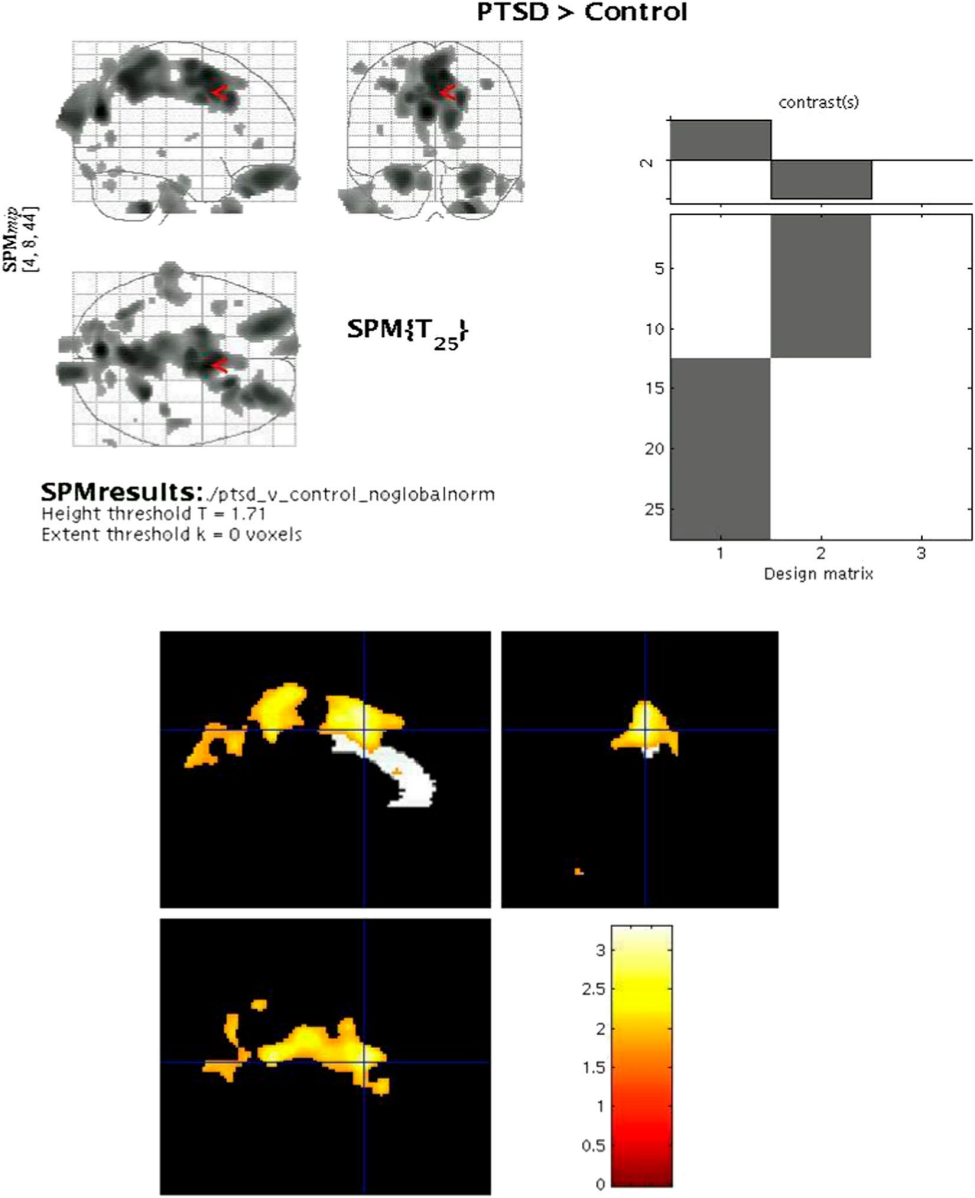
**Brain regions showing a difference in benzodiazepine receptor binding potential (BP) of [** ^**11**^ **C] Flumazenil between post-traumatic stress disorder (PTSD) subjects and healthy controls**

The mean [^11^C]-flumazenil BP was significantly higher in patients with PTSD than in healthy controls in a contiguous cluster comprising the medial and superior portion of the caudal ACC and precuneus (see Table 2 and Fig. 1). No area was identified in which the mean BP was lower in PTSD subjects versus controls.

**Table 2:** Brain regions showing a difference in BZD receptor binding potential (BP) of [^11^C] Flumazenil between PTSD subjects and healthy controls, and brain regions showing a significant positive correlation between BP and PTSD severity (CAPS score) in subjects with PTSD

BP in brain region	*x*, *y*, *z* coordinates	*T*-value	Voxel *p*-value, uncorrected	Cluster size	Cluster *p*-value, corrected
**PTSD** **>** **HC**					
Superior & Caudal anterior cingulate cortex	4, 4, 47	3.12	0.002	8429	0.002
Precuneus	−1, −52, 42	3.03	0.003		
Precuneus	−6, −74, 22	3.30	0.001		
**CAPS-BP correlation**					
Left mid-insula	−32, −14, 8	3.68	0.002	618	N.A.
Left anterior insula	−30, 10, 4	3.32	0.004		

*BP* binding potential, *BZD* benzodiazepines, *PTSD* post-traumatic stress disorder, *HC* healthy controls, *CAPS* clinician-administered PTSD scale

The regional BP values positively correlated with CAPS scores in the left mid-insula (*x* = −32, *y* = −14, *z* = 8; *t* = 3.68; *p*_uncorrected_ = 0.002) and left anterior insula (*x* = −30, *y* = 10, *z* = 4; *t* = 3.33; *p*_uncorrected_ = 0.004). The correlational analyses were conducted only within the PTSD group, as the control subjects were healthy non-trauma-exposed subjects with CAPS scores of 0 (as shown in Table 1).

The injected dose of [^11^C]-flumazenil did not differ significantly between the PTSD and control groups (19.2 (1.1) and 20.4 (0.9) mCi, respectively, *p* = 0.31). At the time of injection, the mean specific activity of the [^11^C]-flumazenil administered was 1562 (+434) mCi/μmol in the PTSD group and 1608 (±301) mCi/μmol in the control group (*p* = 0.36).

## Discussion

Relative to healthy controls, PTSD subjects showed increased BZD receptor BP in the precuneus, and the superior and dorsal ACC. We further report a positive correlation between PTSD symptom severity and BZD BP in the left anterior and mid-insula, which are key regions of the salience network (SN) and cerebral targets of the afferent projections of the vagus nerve, as reviewed below.

The precuneus displays the highest resting metabolic rate in the brain, consuming 35% more glucose than any other cerebral region^20^. It is more highly developed in humans than in non-human primates or other animals, has the most complex columnar cortical organization, and is among the last regions to myelinate^21^. It is also one of the more highly interconnected brain regions, with unilateral and bilateral reciprocal cortico-cortical connections to areas of the posteromedial cortex, other parietal areas, frontal lobe regions, and the basal ganglia^22^, and participates in the neural processing of visuospatial imagery.

The precuneus plays a central role in default mode network (DMN) function^23^. The DMN is an intrinsic or resting-state connectivity network (ICN; RSCN). Such networks are characterized on the basis of functionally connected brain networks that are specifically active during resting state or task-oriented conditions^24^. These large-scale neurophysiological networks are associated with characteristic functions^25^, are stable across tasks^26^ and time^27^, correspond to anatomical white matter tracts^28^, and demonstrate direct behavioral correlates^29^. The DMN is a highly coordinated neuronal network engaged in stimulus-independent activity, and functions to integrate visuospatial imagery, episodic memory retrieval, non-task-related introspection, self-awareness^30^, and monitoring of the environment for potential threats^31^. Deficiencies in DMN functional connectivity were identified as early markers of trauma exposure^32^. Decreased connectivity during subliminal threat processing was found in PTSD compared to controls in the precuneus^33^. In symptom provocation studies, PTSD patients exhibit greater activation than controls in the precuneus^34^. In contrast, in resting-state functional MRI (fMRI) studies, precuneus activation was lower in PTSD and inversely correlated with re-experiencing symptoms^35^, suggesting that dysregulation in the precuneus/DMN “gateway” function of maintaining a resting state may be associated with PTSD pathophysiology.

Reuveni et al.^36^ report a negative correlation between functional connectivity levels and anxiety and depression symptomatology in the precuneus of patients with PTSD. However, no differences in anatomical and functional connectivity patterns were found between chronic, severely-ill PTSD patients and trauma-exposed controls in the precuneus and all other brain regions. Consistent with the results of the present study, this finding illustrates a state wherein an association between clinical symptoms and regional brain function in the PTSD group is not accompanied by a between-group difference in brain function in the same or any other brain region.

A more recent study of connectome-wide investigation of altered resting-state functional connectivity in war veterans with and without PTSD found that veterans without PTSD showed reduced connectivity relative to healthy non-traumatized controls between the precuneus and several other brain areas. The authors hypothesized that the decreased connectivity of the precuneus may represent suppression of the retrieval of sensory memory of traumatic events^37^. Recent studies exhibit differential alteration in the DMN subsystems in PTSD patients compared to controls. Specifically, there were changes in connectivity in the PTSD group involving the medial temporal lobe subsystem with reduced correlation between the posterior cingulate cortex and the hippocampus, and reduced anticorrelation between the ventromedial prefrontal cortex and the dorsal ACC^38^.

The purported role of the DMN is to maintain and integrate endogenous brain activity and exogenous task-related processes. The continuous state of hyperarousal and increased reactivity in PTSD alludes to a flaw in DMN function in this condition. Nevertheless, the neurochemical mechanisms mediating this process have not been elucidated.

GABA-ergic transmission has been implicated in the regulation of RSCNs^39^. Available data are limited, and the relationship between GABA-ergic activity and PTSD symptomatology requires further investigation. A negative correlation between regional cerebral GABA concentrations and both hemodynamic activity and cognitive performance (speed) has repeatedly been shown^39–42^. This inverse association between GABA and DMN deactivation increases with cognitive demand^43^. In an interesting study, Wiebking et al.^44^ used fMRI combined with [^18^F]-flumazenil-PET in healthy subjects to study the effects of monitoring internal (heartbeat counting) and external (tone counting) stimuli upon brain neurotransmitters and hemodynamic activity in cortical midline regions. They found that although both stimulus types induce negative blood-oxygen-level dependent contrast imaging responses in the precuneus, the magnitude of the baseline BZD receptor BP was correlated with the hemodynamic activity changes associated with external, rather than internal, awareness.

A hallmark of PTSD is a continuous high level of arousal and excessive physiological and emotional responses to everyday internal or external stimuli^45–47^. This intrusion of ruminative ideation or emotional responses to external stimuli may impair attentional focus on other cognitive processes. Internally driven symptoms such as intrusive memories and dissociation, hyperarousal, and continued anticipatory anxiety, prominent in PTSD indicate that a “resting” (behaviorally) state is practically nonexistent in PTSD. This observation is compatible with altered BZD receptor function in key nodes of the DMN. The elevation we found in BZD receptor BP may conceivably attest to an altered inhibition exercised upon the precuneus, expressed by the absence of a “resting state” observed in this population.

The BP was also increased in the caudal ACC, a region involved in diverse functions. For example, the region implicated specifically by the peak voxel *t*-values manifests sustained neural activity during working memory tasks, with a pattern of activity suggesting that this region mediates a state of preparedness for selecting a motor response^48^. However, the cluster of voxel *t*-values in this region also extends through mid- and posterior cingulate cortical areas that form part of the extended medial prefrontal network, and participate in anxiety and emotional processing^31,49^, error detection, performance monitoring, competition monitoring, anticipation, working memory, novelty detection, and reward assessment^50^. Impairment in such functions can be readily ascribed to PTSD, in particular in the symptom clusters of cognitive alteration, avoidance, and increased arousal. In fact, hyper-responsivity of the dorsal ACC appears to be a constitutional familial vulnerability factor for the development of PTSD following exposure to threatening events^51,52^. The dorsal ACC has also been implicated in mediating fear extinction learning^53,54^. Greater avoidance symptomatology in PTSD was associated with greater activation in brain regions overlapping with the dorsal ACC during both fear acquisition and extinction^55^. Heightened fear responses and increased avoidance symptoms in individuals with PTSD suggest a deficiency in fear extinction learning. Higher dorsal ACC activity in response to negative images reportedly predicted the persistence of PTSD symptoms measured 6–8 months after treatment^56^, as well as symptom severity at long-term follow-up (4 years)^57^. In light of GABA’s well-established involvement in fear conditioning and extinction mechanisms^58^ and the key role of the dorsal ACC in mediating these processes, our finding of increased flumazenil BP in the dorsal ACC offers an invaluable direction for future research to unravel the underpinnings of impaired fear extinction learning in PTSD.

A positive correlation was found between BZD receptor BP and PTSD severity (CAPS score) in the left mid-insula and left anterior insula, although this finding must be considered exploratory because the *p*-value was not corrected for multiple testing. The insular cortex plays a major role in processing emotional and cognitive information, self-awareness, and anticipation of aversive stimuli^59^. In PTSD, the capacity to identify and differentiate between stimuli is impaired, leading to a generalization of fear responses arising from emotionally neutral stimuli. Given evidence for autonomic arousal in PTSD, it is noteworthy that the mid-insular area in which BZD receptor BP was abnormal is located the vicinity of the efferent terminal fields from the vagus nerve implicated in interoception^60,61^. This area previously showed reduced hemodynamic responses during interoceptive tasks in patients with major depressive disorder (particularly in depressed subjects experiencing appetite loss) versus controls, to an extent that correlated with the severity of both depressive symptoms and somatic symptoms in depressed subjects^62^. Our preliminary observation of a positive correlation between BZD receptor BP in this region and PTSD symptom severity thus warrants further exploration, particularly in relation to the neural processing of interoception, autonomic arousal, and depression within the context of PTSD.

The insula as well as the dorsal ACC also are central to the SN^63^, an ICN that responds to the degree of personal relevance of a given cue and activates strongly after exposure to emotionally laden stimuli^64^. The SN is also involved in decision-making and coordination of behavioral responses, homeostatic regulation, and reward processing^65^. Disruptions in the SN have been shown to correlate with PTSD symptoms such as anxiety and deficient emotional processing during stressful events^66,67^. It has been hypothesized^68^ that PTSD may be characterized by a weakly connected and hypoactive DMN and central executive network (CEN) that are destabilized by an overactive and hyperconnected SN, with a low threshold for perceived saliency and inefficient DMN-CEN modulation.

Relative to healthy controls we found increased basal BZD receptor BP in patients with PTSD in brain regions comprising the DMN and SN. The activity of these networks is considered anticorrelated, subserving different attributes of foci of attention^25^. The SN, involved in the capture of relevant external stimuli, signals the DMN to reduce activity when attention should be externally focused. Damage to white matter tracts within the SN impairs this dynamic network interaction^69^. Sripada et al.^67^ exhibited increased cross-network connectivity between DMN and SN in PTSD, also reported by Zhang et al.^70^. Two studies employing graph theory analysis to analyze resting-state fMRI data suggest that disequilibrium between the DMN and SN is conceivably associated with PTSD pathophysiology and could serve as a biomarker for the disorder^71,72^.

Our findings of increased BZD receptor BP in PTSD differ from the findings of two previous SPECT-[123I]-iomazenil studies^1,3^ and one PET study^56^ in PTSD. These studies limited their patient samples to male combat veterans, and it is conceivable that the distinct findings in the current study are influenced by sex differences in BZD receptor binding. Control subjects in previous studies also varied: trauma-exposed veterans in the PET study of Geuze et al.^2^, non-deployed veterans in one of the SPECT studies^3^, and healthy, non-exposed controls in the other^1^. We studied a combined male and female sample of PTSD subjects who had been exposed to civilian trauma, both childhood and adult, compared to healthy, non-trauma-exposed controls. Moreover, we included only participants who were naïve to psychotropic medication and did not meet criteria for current or past alcohol or substance abuse or dependence. In the previous studies cited above the past psychotropic medication usage also varied, with no usage of BZD in the 6 months preceding the study in all three studies, but with apparent use of other psychotropic medication 4 weeks or more prior to the beginning of the two SPECT studies. Furthermore, although substance and alcohol abuse in the 6 months preceding the evaluation was an exclusion criterion in all three of these previous studies, in the Fujita et al.^3^ study almost one-half of participants had a history of abuse while in the Bremner et al.^1^ and Geuze et al.^2^ studies 23% and 22% (respectively) of participants had a history of abuse.

Notably, a meta-analysis of fMRI data from PTSD samples reported that differences in hemodynamic activity between patients with PTSD and controls varied according to the presence/absence of trauma exposure in the control group. Particularly, the activity in the precuneus differed between groups when PTSD was compared to trauma naïve subjects, as in the present study, but not when compared to trauma-exposed controls^73^. Thus, our findings may reflect differences in subject sample selection, with our PTSD and control sample free from confounding effects of current or past psychotropic drug use or abuse, and the control sample trauma naïve, and/or in their methods for measuring BZD receptor BP, since the PET-[^11^C]-flumazenil technique has superior sensitivity and relative to SPECT-[^123^I]-iomazenil.

The data suggest a deficit in GABA-ergic transmission in PTSD in the precuneus and posterior cingulate regions, resulting in compensatory upregulation of BZD receptors. Potentially consistent with this hypothesis, BZD receptor agonists are often used to treat anxiety symptoms in PTSD. However, the efficacy of BZDs on the core symptoms of PTSD has remained debatable^74^, with potential complications of dependence, withdrawal, as well as excessive sedation, reduced concentration, and attention^75^. Other compounds that modulate GABA_A_ receptor function thus have been explored. Decreased levels of allopregnanolone (Allo), a potent intrinsic modulator of GABA_A_ receptors^76^, have been associated with development of PTSD in women^77^. Studies using socially isolated mice, as an animal model of PTSD, have also demonstrated that corticolimbic Allo levels become markedly decreased in association with the development of anxiety-like behaviors, resistance to sedation, and extreme aggression^78–80^. Hence, treatment with pregnenolone appears to have the potential to improve emotional regulation by increasing Allo levels and reducing activation of brain areas involved in mediating negatively valenced emotions^81^. Nevertheless, a phase 2 clinical trial of ganaxolone, a synthetic analog of Allo, in PTSD patients showed no benefit relative to placebo in reducing PTSD symptoms^82^.

## References

[1] Bremner JD (2000). Decreased benzodiazepine receptor binding in prefrontal cortex in combat-related posttraumatic stress disorder. Am. J. Psychiatry.

[2] Geuze E (2008). Reduced GABAA benzodiazepine receptor binding in veterans with post-traumatic stress disorder. Mol. Psychiatry.

[3] Fujita M (2004). Central type benzodiazepine receptors in Gulf War veterans with posttraumatic stress disorder. Biol. Psychiatry.

[4] Rossi S (2009). Dysfunctions of cortical excitability in drug-naïve posttraumatic stress disorder patients. Biol. Psychiatry.

[5] Meyerhoff DJ, Mon A, Metzler T, Neylan TC (2014). Cortical gamma-aminobutyric acid and glutamate in posttraumatic stress disorder and their relationships to self-reported sleep quality. Sleep.

[6] Michels L (2014). Prefrontal GABA and glutathione imbalance in posttraumatic stress disorder: preliminary findings. Psychiatry Res..

[7] Rosso IM (2014). Insula and anterior cingulate GABA levels in posttraumatic stress disorder: preliminary findings using magnetic resonance spectroscopy. Depress Anxiety.

[8] Schür RR (2016). Brain GABA levels across psychiatric disorders: a systematic literature review and meta-analysis of (1) H-MRS studies. Hum. Brain Mapp..

[9] Dyke K (2017). Comparing GABA-dependent physiological measures of inhibition with proton magnetic resonance spectroscopy measurement of GABA using ultra-high-field MRI. Neuroimage.

[10] Myers JFM, Evans CJ, Kalk NJ, Edden RAE, Lingford-Hughes AR (2014). Measurement of GABA using J-difference edited 1H-MRS following modulation of synaptic GABA concentration with tiagabine. Synapse.

[11] First M., Spitzer R., Gibbon, M. & Williams, J.B.W. *Structured Clinical Interview for DSM-IV Axis Disorders—Patient Edition (SCID-I/P Version 2.0)* (New York Biometrics Res Dep New York State Psychiatr Inst., 1996)

[12] Blake DD (1995). The development of a Clinician-Administered PTSD Scale. J. Trauma. Stress.

[13] Rush AJ, Gullion CM, Basco MR, Jarrett RB, Trivedi MH (1996). The Inventory of Depressive Symptomatology (IDS): psychometric properties. Psychol. Med..

[14] Hamilton M (1959). The assessment of anxiety states by rating. Br. J. Med. Psychol..

[15] Wu Y, Carson RE (2002). Noise reduction in the simplified reference tissue model for neuroreceptor functional imaging. J. Cereb. Blood Flow. Metab..

[16] Millet P, Graf C, Buck A, Walder B, Ibáñez V (2002). Evaluation of the reference tissue models for PET and SPECT benzodiazepine binding parameters. Neuroimage.

[17] Klumpers UMH (2008). Comparison of plasma input and reference tissue models for analysing [(11)C]flumazenil studies. J. Cereb. Blood Flow Metab..

[18] Poline JB, Worsley KJ, Evans AC, Friston KJ (1997). Combining spatial extent and peak intensity to test for activations in functional imaging. Neuroimage.

[19] Talairach, J. & Tournoux, P. *Co-planar Stereotaxic Atlas of the Human Brain* Vol. 270, 132 (Theime, Stuttgart, Germany, 1988).

[20] Gusnard DA, Raichle ME, Raichle ME (2001). Searching for a baseline: functional imaging and the resting human brain. Nat. Rev. Neurosci..

[21] Goldman-Rakic PS (1987). Development of cortical circuitry and cognitive function. Child Dev..

[22] Cavanna AE, Trimble MR (2006). The precuneus: a review of its functional anatomy and behavioural correlates. Brain.

[23] Utevsky AV, Smith DV, Huettel SA (2014). Precuneus is a functional core of the default-mode network. J. Neurosci..

[24] Raichle ME (2015). The restless brain: how intrinsic activity organizes brain function. Philos. Trans. R. Soc. Lond. B Biol. Sci..

[25] Fox MD (2005). The human brain is intrinsically organized into dynamic, anticorrelated functional networks. Proc. Natl Acad. Sci. USA.

[26] Laird AR (2011). Behavioral interpretations of intrinsic connectivity networks. J. Cogn. Neurosci..

[27] Zuo XN (2010). Reliable intrinsic connectivity networks: test-retest evaluation using ICA and dual regression approach. Neuroimage.

[28] Van den Heuvel MP, Mandl RCW, Kahn RS, Hulshoff Pol HE (2009). Functionally linked resting-state networks reflect the underlying structural connectivity architecture of the human brain. Hum. Brain. Mapp..

[29] Hamilton JP (2011). Default-mode and task-positive network activity in major depressive disorder: implications for adaptive and maladaptive rumination. Biol. Psychiatry.

[30] Whitfield-Gabrieli S, Ford JM (2012). Default mode network activity and connectivity in psychopathology. Annu. Rev. Clin. Psychol..

[31] Price JL, Drevets WC (2012). Neural circuits underlying the pathophysiology of mood disorders. Trends Cogn. Sci..

[32] Zhou Y (2012). Default-mode network disruption in mild traumatic brain injury. Radiology.

[33] Rabellino D (2015). Intrinsic Connectivity Networks in post-traumatic stress disorder during sub- and supraliminal processing of threat-related stimuli. Acta Psychiatr. Scand..

[34] Sartory G (2013). In search of the trauma memory: a meta-analysis of functional neuroimaging studies of symptom provocation in posttraumatic stress disorder (PTSD). PLoS ONE.

[35] Yan X (2013). Spontaneous brain activity in combat related PTSD. Neurosci. Lett..

[36] Reuveni I (2016). Anatomical and functional connectivity in the default mode network of post-traumatic stress disorder patients after civilian and military-related trauma. Hum. Brain. Mapp..

[37] Misaki M (2018). Connectome-wide investigation of altered resting-state functional connectivity in war veterans with and without posttraumatic stress disorder. Neuroimage Clin..

[38] Miller DR (2017). Default mode network subsystems are differentially disrupted in posttraumatic stress disorder. Biol. Psychiatry Cogn. Neurosci. Neuroimaging..

[39] Northoff G (2007). GABA concentrations in the human anterior cingulate cortex predict negative BOLD responses in fMRI. Nat. Neurosci..

[40] Muthukumaraswamy SD, Edden RAE, Jones DK, Swettenham JB, Singh KD (2009). Resting GABA concentration predicts peak gamma frequency and fMRI amplitude in response to visual stimulation in humans. Proc. Natl Acad. Sci. USA.

[41] Sumner P, Edden RAE, Bompas A, Evans CJ, Singh KD (2010). More GABA, less distraction: a neurochemical predictor of motor decision speed. Nat. Neurosci..

[42] Kapogiannis D, Reiter DA, Willette AA, Mattson MP (2013). Posteromedial cortex glutamate and GABA predict intrinsic functional connectivity of the default mode network. Neuroimage.

[43] Hu Y, Chen X, Gu H, Yang Y (2013). Resting-state glutamate and GABA concentrations predict task-induced deactivation in the default mode network. J. Neurosci..

[44] Wiebking C (2014). External awareness and GABA-A multimodal imaging study combining fMRI and [1^8^ F]flumazenil-PET. Hum. Brain. Mapp..

[45] Bauer ME, Wieck A, Lopes RP, Teixeira AL, Grassi-Oliveira R (2010). Interplay between neuroimmunoendocrine systems during post-traumatic stress disorder: a minireview. Neuroimmunomodulation.

[46] MJAG Henckens (2009). memories: how acute stress affects memory formation in humans. J. Neurosci..

[47] Kemp AH (2009). Heterogeneity of non-conscious fear perception in posttraumatic stress disorder as a function of physiological arousal: an fMRI study. Psychiatry Res..

[48] Petit L, Courtney SM, Ungerleider LG, Haxby JV (1998). Sustained activity in the medial wall during working memory delays. J. Neurosci..

[49] Maddock RJ, Casazza GA, Fernandez DH, Maddock MI (2016). Acute modulation of cortical glutamate and GABA content by physical activity. J. Neurosci..

[50] Bush G (2002). Dorsal anterior cingulate cortex: a role in reward-based decision making. Proc. Natl Acad. Sci. USA.

[51] Shin LM (2011). Exaggerated activation of dorsal anterior cingulate cortex during cognitive interference: a monozygotic twin study of posttraumatic stress disorder. Am. J. Psychiatry.

[52] Admon R, Milad MR, Hendler T (2013). A causal model of post-traumatic stress disorder: disentangling predisposed from acquired neural abnormalities. Trends Cogn. Sci..

[53] Milad MR, Quirk GJ (2012). Fear extinction as a model for translational neuroscience: ten years of progress. Annu. Rev. Psychol..

[54] Shvil E (2014). Sex differences in extinction recall in posttraumatic stress disorder: a pilot fMRI study. Neurobiol. Learn. Mem..

[55] Sripada RK, Garfinkel SN, Liberzon I (2013). Avoidant symptoms in PTSD predict fear circuit activation during multimodal fear extinction. Front. Hum. Neurosci..

[56] van Rooij SJH, Kennis M, Vink M, Geuze E (2016). Predicting treatment outcome in PTSD: a longitudinal functional MRI study on trauma-unrelated emotional processing. Neuropsychopharmacology.

[57] Kennis M, van Rooij SJH, Reijnen A, Geuze E (2017). The predictive value of dorsal cingulate activity and fractional anisotropy on long-term PTSD symptom severity. Depress Anxiety.

[58] Singewald N, Schmuckermair C, Whittle N, Holmes A, Ressler KJ (2015). Pharmacology of cognitive enhancers for exposure-based therapy of fear, anxiety and trauma-related disorders. Pharmacol. Ther..

[59] Gasquoine PG (2014). Contributions of the insula to cognition and emotion. Neuropsychol. Rev..

[60] Simmons WK (2013). Keeping the body in mind: insula functional organization and functional connectivity integrate interoceptive, exteroceptive, and emotional awareness. Hum. Brain. Mapp..

[61] Avery JA (2014). Major depressive disorder is associated with abnormal interoceptive activity and functional connectivity in the insula. Biol. Psychiatry.

[62] Simmons WK (2016). Depression-related increases and decreases in appetite: dissociable patterns of aberrant activity in reward and interoceptive neurocircuitry. Am. J. Psychiatry.

[63] Seeley WW (2007). Dissociable intrinsic connectivity networks for salience processing and executive control. J. Neurosci..

[64] Somerville LH, Whalen PJ, Kelley WM (2010). Human bed nucleus of the stria terminalis indexes hypervigilant threat monitoring. Biol. Psychiatry.

[65] Menon V, Uddin LQ (2010). Saliency, switching, attention and control: a network model of insula function. Brain. Struct. Funct..

[66] Patel R, Spreng RN, Shin LM, Girard TA (2012). Neurocircuitry models of posttraumatic stress disorder and beyond: a meta-analysis of functional neuroimaging studies. Neurosci. Biobehav. Rev..

[67] Sripada RK (2012). Neural dysregulation in posttraumatic stress disorder: evidence for disrupted equilibrium between salience and default mode brain networks. Psychosom. Med..

[68] Akiki TJ, Averill CL, Abdallah CG (2017). A network-based neurobiological model of PTSD: evidence from structural and functional neuroimaging studies. Curr. Psychiatry Rep..

[69] Jilka SR (2014). Damage to the salience network and interactions with the default mode network. J. Neurosci..

[70] Zhang Y (2015). Intranetwork and internetwork functional connectivity alterations in post-traumatic stress disorder. J. Affect Disord..

[71] Suo X (2015). Disrupted brain network topology in pediatric posttraumatic stress disorder: a resting-state fMRI study. Hum. Brain Mapp..

[72] Lei D (2015). Disrupted functional brain connectome in patients with posttraumatic stress disorder. Radiology.

[73] Stark EA (2015). Post-traumatic stress influences the brain even in the absence of symptoms: a systematic, quantitative meta-analysis of neuroimaging studies. Neurosci. Biobehav. Rev..

[74] Guina J, Rossetter SR, Derhodes BJ, Nahhas RW, Welton RS (2015). Benzodiazepines for PTSD: a systematic review and meta-analysis. J. Psychiatr. Pract..

[75] Soyka M (2017). Treatment of benzodiazepine dependence. N. Engl. J. Med..

[76] Belelli D, Lambert JJ (2005). Neurosteroids: endogenous regulators of the GABAA receptor. Nat. Rev. Neurosci..

[77] Rasmusson AM (2006). Decreased cerebrospinal fluid allopregnanolone levels in women with posttraumatic stress disorder. Biol. Psychiatry.

[78] Nin M, Martinez LA, Pibiri F, Nelson M, Pinna G (2011). Neurosteroids reduce social isolation-induced behavioral deficits: a proposed link with neurosteroid-mediated upregulation of BDNF expression. Front. Endocrinol. (Lausanne).

[79] Pibiri F, Nelson M, Guidotti A, Costa E, Pinna G (2008). Decreased corticolimbic allopregnanolone expression during social isolation enhances contextual fear: A model relevant for posttraumatic stress disorder. Proc. Natl Acad. Sci. USA.

[80] Pinna G, Rasmusson AM (2012). Up-regulation of neurosteroid biosynthesis as a pharmacological strategy to improve behavioural deficits in a putative mouse model of post-traumatic stress disorder. J. Neuroendocrinol..

[81] Sripada RK (2013). Allopregnanolone elevations following pregnenolone administration are associated with enhanced activation of emotion regulation neurocircuits. Biol. Psychiatry.

[82] Rasmusson AM (2017). A randomized controlled trial of ganaxolone in posttraumatic stress disorder. Psychopharmacology (Berlin).

